# Experiences of migrant mothers attending vaccination services at primary healthcare facilities

**DOI:** 10.4102/hsag.v28i0.2166

**Published:** 2023-05-19

**Authors:** Stephan Acheampong, Mygirl P. Lowane, Lucy Fernandes

**Affiliations:** 1 Department of Public Health, School of Healthcare Sciences, Sefako Makgatho Health Sciences University, Ga-Rankuwa, South Africa

**Keywords:** Migrant mothers, utilisation, immunisation services, primary healthcare, vaccines, language difficulties, interpersonal relationship, healthcare workers

## Abstract

**Background:**

Migration to South Africa is currently dominated by women and children, for socio-economic and refugee reasons or to utilise the healthcare system for various services. Migrants and refugees are at risk of vaccine-preventable diseases, and many of their children have an incomplete or unknown immunisation status.

**Aim:**

This study aimed to explore the experiences of migrant mothers in utilising child immunisation services in primary healthcare facilities.

**Setting:**

Ten primary healthcare facilities that were providing immunisation services, located in the Buffalo City Metropolitan Municipality, Eastern Cape province, South Africa.

**Methods:**

A qualitative research design, making use of in-depth interviews (IDIs) from 18 purposefully selected migrant women, was used for data collection. Thematic content analysis was used to analyse the recorded data of the experiences of study participants in their access to immunisation services.

**Results:**

From the IDIs, four themes were identified: difficulty in communicating with the healthcare workers because of language barriers, access challenges, interpersonal barriers and interpersonal relationships were identified in this study, which influenced the utilisation of immunisation services by migrant mothers.

**Conclusion:**

The findings of this study support and reinforce the duty of the South African government and healthcare facilities to work together to improve migrant women’s access to immunisation services.

**Contribution:**

A positive relationship between healthcare workers and migrant mothers while accessing immunisation services should contribute to reducing child mortality in South Africa and achieving Sustainable Development Goal 3 by the year 2030.

## Introduction

Men previously dominated migration into South Africa. In recent years, there has been an increase in the migration of women (Crush, Williams & Peberdy 2005; Von Fintel & Moses 2017). Marnell, Oliveira and Khan (2021) report that women migrants in well-developed and less-developed countries make up 50% and 45.7% of migrants, respectively, representing 3.3% of the world’s population. Compared to the global percentage, Makandwa and Vearey (2017) report that migrant women within the Southern African Development Community (SADC) subregion make up less than 45% and 42.7% in South Africa, respectively. Several factors, such as socio-economic, demographic factors and political instability, were found to be reasons for most cross-border migrations (Saburi 2017).

Utilisation of healthcare services by migrant women has recently been perceived to be an issue in the public healthcare system (Machiwenyika & Munatswa 2020). Chekero and Ross (2018) mentioned that pregnant women and children under 6 years have free access to healthcare, regardless of migration status. Machiwenyika and Munatswa (2020) reported that because of increased trends in migration, utilisation of healthcare services by migrant mothers has become controversial and a political issue.

Women are important contributors to the uptake of childhood immunisation (Gorman et al. 2019). Immunisation service is one of the most cost-effective maternal healthcare interventions which reduces child mortality and burden of childhood illnesses (Akwataghibe et al. 2019; Charania et al. 2018). As migrant women move from one place to another, some give birth in a foreign country (Saburi 2017). Porth et al. (2021) admonish that maternal migration may result in immunisation disparities and that social determinants of health and societal attitudes determine migrants mothers’ experience of utilising healthcare services. Machiwenyika and Munatswa (2020) highlighted that migrants suffer unfair treatment in the new society, even in the healthcare sector. The provision of healthcare services remains a challenge. To promote equity for immunisation services in the healthcare system, availability, accessibility, affordability, adequacy and acceptability should be applied, as they influence health-seeking behaviour (Ma et al. 2018).

### Problem statement and purpose

Infant immunisation helps to strengthen immunity among children, and it is considered the most effective way to reduce childhood illness and deaths. Immunisation is regarded as one of the most important achievements of public health and prevents nearly 2–3 million child deaths every year (Han et al. 2014). Several challenges were reported globally; however, Makamba-Mutevedzi, Madhi and Burnett (2020) reported that healthcare facilities’ obstacles in South Africa were the major reasons for low immunisation coverage. Vearey et al. (2018) reported on the diverse challenges of experiencing discrimination and abuse by healthcare providers, yet literature related to the experiences of migrant women in accessing immunisation services in primary healthcare (PHC) facilities is limited. This article aims to explore the experience of migrant mothers accessing immunisation services provided in PHC facilities.

## Research methods

### Study design and setting

A qualitative research design was used to explore the utilisation of immunisation services provided in PHC facilities by migrant mothers. The study was conducted in Buffalo City Metropolitan Municipality, situated on the east coast of Eastern Cape province, South Africa. The Buffalo City Metropolitan Municipality consists of three large towns, namely East London, Bisho and King William’s Town, and two large townships, namely Mdantsane and Zwelitsha. Seethal, Nel and Bwalya (2021) reported that the municipality has an estimated population of 893 157 people. It also has an estimated 135 000 (49.72%) total households, where 70 700 (26.03%) are formal dwelling units and 18 300 (6.73%) are informal dwelling units. The municipality has an estimated population of 701 873, which is largely African (85.2%), with white (8.4%) and mixed-race (5.7%) people in the minority. The leading economic drivers of Buffalo City Metro are community services, which are largely government services, contributing 18.4%, followed by trade (exports of goods and services) at 13.7%, finance at 13.2% and manufacturing at 10.0%. In Buffalo City Metro, the economic sectors that recorded the largest employment numbers in 2018 were the community services sector, with a total of 64 600 employed people or 25.5% of total employment in the metropolitan municipality. A large percentage of Buffalo City Metro road infrastructure is old and rapidly deteriorating, having passed its design life. The metropolitan municipality has 79 PHC facilities. The East London suburbs were considered because of their dense population of migrants, many running small businesses. Ten facilities with a high volume of migrant mothers accessing the facilities were selected for the study.

### Study population, inclusion criteria and sampling

Migrant mothers, aged 18 years and older, with children aged 0–23 months, were purposively selected to participate in this study. Only mothers who could communicate in the English language were selected. Mothers with a record of frequently using the facility for their children’s immunisations and who consented to participate were considered.

### Data collection tool and procedure

A semistructured interview guide, which was adapted from the article by Makandwa (2014), was used for the individual in-depth interviews (IDIs). Open-ended semistructured interviews were used because it is the most effective method for data collection, where the researchers use open-ended data to explore the participants’ views, beliefs and feelings to answer the research question (Newcomer, Hatry & Wholey 2015). The English language was used for data collection. The principal researcher engaged with the healthcare facility supervisors in each PHC facility to arrange for the recruitment of study participants and a safe room for interviews. The principal researcher liaised with the healthcare worker responsible for immunisation services to refer the mothers meeting the inclusion criteria and those willing to participate voluntarily to the place where the IDI was conducted. Information regarding the study was explained to the individual mother by the principal researcher. The principal researcher obtained informed consent, and voluntary participation was ensured. Written informed consent forms were signed by the study participants before the interview commenced. After receiving the signed consent form from the participant, permission to use the digital voice recorder was asked of the participants, and it was used to record the interviews. The principal researcher took the field notes and conducted the interviews. Data were collected over a period of 2 months (October and November 2021). Data saturation was reached after 16 study participants were interviewed. However, the principal researcher interviewed two more study participants to make sure that there was no new information and to declare data saturation.

### Data analysis

The audio-recorded data from the IDIs were transcribed verbatim by the principal researcher on the day the interview was conducted. The transcripts were read several times to initiate the development of codes to develop an accurate codebook. The codebook was developed for coding the data. Thematic analysis was employed while analysing the data. The transcripts were populated into NVivo 12 (QSR International, Burlington, Massachusetts, United States) for analysis. Themes and subthemes were applied to the collected data of all the transcripts in the process of coding. The sociodemographic data of the migrant mothers were summarised in frequency tables.

### Data quality

Credibility, dependability, conformability and transferability were ensured for trustworthiness. The study achieved credibility by ensuring that audio-recorded data from the IDIs were transcribed verbatim and were a true reflection of what the study participants answered in the interviews. Verbatim-transcribed data from the recordings, field notes and observations were kept for audit by the principal researcher for the purpose of verification. Data will be kept for a period of 5 years on Google Cloud (Alphabet, Inc., Mountain View, California, United States) and a hard drive for backup and will be password protected. Dependability was addressed through code-recoding, where consensus was reached with the other researchers. The researchers ensured confirmability by ensuring that the findings were based on participants’ responses instead of their (researchers’) own preconceptions and biases, as well as through audit trials. Furthermore, conformability was achieved by applying objectivity during data collection and analysis. Transferability was ensured by discussing the results of the study in depth with supporting direct quotations from the interviews. All three authors took part in the data analysis process.

### Ethical considerations

This article originated from the first author’s mini-dissertation, which was submitted in partial fulfilment of the requirement of a Master’s in Public Health. Sefako Makgatho Health Sciences University Research and Ethical Committee (reference number SMUREC/H/61/2021:PG) granted the ethical clearance for this study. Permission was granted by the Eastern Cape Department of Health Research Committee to conduct the study at the various healthcare facilities and clinics in Buffalo City Metropolitan Municipality. Voluntary consent was obtained from the mothers, and interviews were conducted without any coercion. Confidentiality, justice and anonymity were assured, and pseudonyms were used during the interviews to protect the participants’ identity. Participants were informed about the option of withdrawal from participating in the study at any point without any consequences.

## Results

Eighteen mothers participated in this study. Seventeen out of 18 participants had Home Affairs documents with them during data collection (see Table 1). Ten participants have been in South Africa for 5 years or less; 12 were employed; 11 were between 25 and 34 years old, and six were above 35 years old. The sociodemographic characteristics of the participants are presented in Table 1.

**TABLE 1:** Sociodemographic characteristics of migrant mothers (*N* = 18).

Characteristics	Variable	Frequency
Registration with the Department of Home Affairs	Asylum seeker	9
	Work permit	7
	Study permit	1
	No registration	1
**Employment status**		
Employed (*N* = 12)	Self-employed	4
	Casual	1
	Unskilled	2
	Temporary	2
	Domestic	3
Unemployed		6
Age (years)	18–24	1
	25–34	11
	35–44	5
	45–49	1
Number of years living in South Africa	1–5	10
	6–10	4
	11–15	4

### Themes and subthemes identified

From the IDIs, four themes were identified relating to the experiences of migrant mothers at the PHC facility that they utilised for child vaccination. A summary of the main themes and subthemes that emerged from the data analysis is presented in Table 2.

**TABLE 2:** Summary of themes and subthemes.

Themes	Subthemes
**1. Communication difficulties**	1a. Language barriers
2. Barriers to accessing immunisation services as perceived by migrant mothers	2a. Healthcare service accessibility
	2b. Vaccination times and delays at the healthcare facility
3. Socio-economic factors hindering utilisation of immunisation services at healthcare facilities	3a. Nature of migrant employment status
	3b. Insufficient funds to travel to a healthcare facility
4. Interpersonal relationships with healthcare staff	4a. Equal treatment based on nationality
	4b. Health workers’ attitudes towards immigrant mothers
	4c. Perceived discrimination by health workers

#### Theme 1: Communication difficulties

Some migrant mothers found it difficult to communicate with the healthcare workers, as they were not able to speak English for daily conversation and could not converse in medical terms. Almost all the participating migrant mothers reported difficulty communicating in any of the local languages:

‘I can speak isiXhosa and English a bit, but to understand medical terms for me is too difficult.’ (IDI 10, Mini, 19 years old)

‘Understanding medical conversation with the nurses is much more difficult than literal understanding of the conversation.’ (IDI 6, Zeidah, 32 years old)

The fact that they were not able to communicate with the nurses means that some had to bring a person with them who could help them interpret the conversations taking place at the healthcare facilities. Three participants mentioned that they rely on their spouses for interpretations during healthcare facility visits:

‘I can’t speak English, so I ask my husband to accompany me if he is available that day. If he went to work I cannot go to the healthcare facility, because he is the one who’s going to be an interpreter.’ (IDI 1, Mindo, 27 years old)

**Subtheme 1a: Language barriers:** Some participants felt that the service rendered is compromised because they cannot express themselves, raise concerns or ask questions regarding the vaccination. Most participants indicated that they cannot speak either isiXhosa or English fluently, so they felt that nurses were also frustrated because they likewise could not reply in the language of the migrant:

‘Yeah … I cannot speak their [*nurses’*] language, so I think they [*nurses*] must be also frustrated because they can’t express themselves to us when they want to give health promotion messages.’ (IDI 5, Rose, 33 years old)

A few participants reported being able to communicate in English, but they still encounter challenges because some nurses refuse to communicate in English during the consultations:

‘Yeah … sometimes like when you can’t speak the isiXhosa and the nurse doesn’t want to speak in English. It affects the quality of service because you don’t understand what they are saying to you.’ (IDI 4, Nomzamo, 30 years old)

#### Theme 2: Barriers to access immunisation services as perceived by migrants mothers

Migrant mothers experienced difficulty accessing healthcare facilities. The distance to the healthcare facility and the times at which the services were offered were the major barriers affecting those mothers’ use of child immunisation services.

**Subtheme 2a: Healthcare service accessibility:** Most migrant mothers reported staying far away from the healthcare facility, which made it difficult for them to access the healthcare facility for immunisation services for their children, as evidenced by the following quotes:

‘Umm … where I’m staying is very far, we take two taxis to come here in town to this healthcare facility.’ (IDI 6, Zaidah, 32 years old)

Two participants mentioned that sometimes they arrive after 10:00 for the immunisation services and they are turned away to come back the next morning. They suggested that mobile healthcare facilities could be the solution to their challenges:

‘Also, if they can open a healthcare facility in the area where I stay, then we will not travel to come here in town to this healthcare facility. It’s too far to come here.’ (IDI 3, Zeldah, 28 years old)

‘This healthcare facility … immunisation service closes at 11 o’clock every day. The healthcare workers say that vaccines must be out of the refrigerators for only four hours. After 11 [*time*], they tell us to come back the next morning.’ (IDI 16, Anatswanashe, 46 years old)

**Subtheme 2b: Vaccination times and delays at healthcare facilities:** Some participants reported delays in vaccination starting time at some PHC facilities. They reported that this situation affects them because they may leave the facility without their children being vaccinated. They also are not able to go back to work on time:

‘[…*T*]hey usually start immunisation services after some hours after they [*healthcare workers*] arrived to work. Sometimes they [*healthcare workers*] start the immunisation service after nine o’clock. I think the time I spent here is too much because I arrive very early. I leave my job and come here the whole day. Where am I going to get money to buy food for my baby and myself if I spent the whole day at the healthcare facility without working?’ (IDI 8, Fatu, 30 years old)

#### Theme 3: Socio-economic factors hindering utilisation of immunisation services to healthcare facilities

Most migrant mothers encountered some personal barriers in the process of accessing immunisation services for their children. The nature of employment affected some mothers in honouring their immunisation appointment schedules. Others found it difficult to access healthcare facilities because of the cost of travelling expenses, which were not affordable to them.

**Subtheme 3a: Nature of migrant employment status:** Working migrant mothers found it difficult to get sufficient time off to take their children for immunisation services:

‘I am a domestic worker, working Monday to Friday. When I ask to take my child to the healthcare facility for immunisation, sometimes, she [*employer*] will give me permission from morning until ten o’clock. Meanwhile in most cases the immunisation services start after eight o’clock in the healthcare facility.’ (IDI 11, Anishe, 29 years old)

Some participants reported that they are casual and unskilled workers, offering their services on a temporary or casually seasonal basis. Some said that they worked for more than 40 h a week, whereas 2 out of the 12 working participants reported that they are self-employed:

‘You see, sometimes it is difficult to take the time off because I must cover the required hours per week. If I don’t cover those hours, they [*employer*] deduct lot of money from what I was supposed to get.’ (IDI 7, Bernice, 40 years old)

‘Nowadays business is not good so you will go to the healthcare facility the whole day, it means no money for that day.’ (IDI 8, Fatu, 30 years old)

**Subtheme 3b: Insufficient funds to travel to healthcare facilities:** Unemployed migrant mothers found it difficult to access PHC facilities because of financial constraints, and they relied on borrowing money either from their families or their friends to cover the transport cost. Some participants reported that they sometimes resort to walking to the healthcare facilities if they do not have money. This results in them skipping scheduled immunisation schedules. The following is some of the frustration expressed by one participant:

‘With regards to the taxi fare, sometimes I borrow money from my friends and if I don’t get it then that day, I don’t bring my child. I will wait to get money first before I bring the child.’ (IDI 2, Joyce, 32 years old)

#### Theme 4: Interpersonal relationship between healthcare staff and migrant mothers

This theme reports the relationship between migrant mothers and healthcare workers when utilising immunisation services. Three subthemes emerge from this theme.

**Subtheme 4a: Equal treatment irrespective of nationality:** Although the two participating mothers were not happy about the treatment experienced in the healthcare facilities (refer to subthemes 4b and 4c), most migrant mothers were very happy with the treatment that they received at the specific healthcare facility. A majority of participants reported that they received equal and fair treatment from the healthcare workers. One mother has been coming to the healthcare facility for many years, specifically because of the good treatment that she is receiving. Others come to the healthcare facility as they feel there is no segregation of foreigners:

‘The care is amazing, I wouldn’t complain. Because the service that I get is the same as the service South Africans get. I don’t feel left out at all.’ (IDI 16, Anatswanashe, 46 years old)

‘Here they [*healthcare workers*] are treating us nicely and fine. Umm there is no difference between us and the citizens.’ (IDI 17, Mary, 29 years old)

**Subtheme 4ba: Health workers’ attitudes towards immigrant mothers:** Study participants raised issues regarding the attitudes of health workers: both positive and negative attitudes were reported. Their positive comments included politeness and a welcoming attitude:

‘She [*healthcare worker*] is caring. It does not matter to her that I’m from another country. She is very polite. We actually talk about anything, including other services offered in the facility.’ (IDI 18, Anesu, 35 years old)

‘The nurse received me well and took my child for weighing. Everything went well.’ (IDI 12, Mavis, 42 years old)

A few study participants reported uncaring attitudes or suboptimal treatment from health workers. The migrant mothers stated that the attitude of healthcare staff towards them was inappropriate:

‘They are very rude especially when you do not understand their [*healthcare workers’*] language.’ (IDI 16, Beatrice, 26 years old)

‘They [Healthcare workers] must be tolerant to foreigners when we come here and stop shouting at us.’ (IDI 13, Anaishe, 29 years old)

One study participant reported that they were forced to wait outside despite the cold weather. She mentioned that the nurses would call other people to enter first, and she was attended to after a long time:

‘I did not like their treatment, because i was told to wait outside in a cold and drizzling weather, because they [*healthcare workers*] were still preparing the immunisation room.’ (IDI 13, Anaishe, 29 years old)

**Subtheme 4c: Perceived discrimination by health workers:** Six migrant mothers had negative experiences such as discrimination and inappropriate behaviour of the healthcare staff towards them. Two participants reported that they were told that there was a shortage of vaccines, but the healthcare workers immunised other children, not the migrant children:

‘We were told that there are limited vaccine stocks, for that day, they will only immunise the children from this area [*Buffalo City*]. So we were asked to come next week to check whether they have enough stock [*of vaccines*].’ (IDI 7, Bernice, 40 years old)

Other study participants (10) felt that the treatment received showed no discrimination, because the healthcare staff were polite towards them and all children were immunised. They reported that whenever there was limited stock of vaccines, the first-come-first-served rule applied to all mothers bringing their children for immunisation. Migrant mothers also reported that they were not discriminated against even though they went to other healthcare facilities for child vaccinations, in case there was a shortage of vaccines in the usual healthcare facility:

‘They [*nurses*] don’t discriminate and say you are a foreigner or citizen. The experiences and the care they provide here [*healthcare facility*] is the same.’ (IDI 15, Masala, 44 years old)

‘They are treating us the same as South Africans. There is no discrimination here since we are coming from … Normally one might assume we were not going to be treated the same. Well! … but that’s not the case here [*healthcare facility*].’ (IDI 16, Beatrice, 26 years old).

## Discussion

The study aimed to explore the experience of migrant mothers attending vaccination services at PHC facilities. Healthcare utilisation studies among migrant patients are useful to explore migrants’ satisfaction with the health system (White, Levin & Rispel 2020). The Constitution of South Africa stipulates that all people are entitled to utilise healthcare services, regardless of citizenship (White et al. 2020). This practice was also supported by Makandwa (2014) from a study conducted in sub-Saharan Africa. Despite free access to healthcare services, the study identified some negative issues migrant mothers face when utilising healthcare services.

The language barrier was one of the challenges reported by the migrant mothers in this study. Davies, Basten and Frattini (2009), and Makandwa (2014) also reported that language is frequently cited as a major obstacle to the use of healthcare services, as there are no interpreters at the healthcare facilities. Kuan, Chen and Lee (2020) state that the influx of migrants who cannot communicate in English increases the demand for professional interpreters in healthcare facilities. It was also found in a study conducted by Amukugo, Nangombe and Karera (2020) from Namibia that the language barrier is complex in the healthcare process, more especially among healthcare providers who cannot communicate in the English language (Saburi 2017). This situation impacts effective communication with patients and results in difficulty providing good, appropriate care and health education. Good communication reduces anxiety, guilt and confusion among patients, and increases patients’ satisfaction, acceptance and compliance with the healthcare advice and treatment (Amukugo et al. 2020).

Migrant mothers participating in this study raised issues related to access to immunisation services. Some healthcare facilities were located far from where most migrant families lived, which made it difficult for them to access the immunisation service. A similar study conducted in the Philippines by Chung et al. (2016) found that the distance to the various healthcare facilities was significantly associated with poor immunisation uptake among migrant mothers. Tankwanchi et al. (2021) report that the health of refugees and migrants was given priority at the 72nd World Health Assembly of 2019, because it was identified that many immigrant communities experience lower immunisation rates and a higher burden of vaccine-preventable diseases when compared to the host country. The researchers in this study found no study that analysed migration as a factor for child immunisation uptake in South Africa.

However, participants in the present study reported long waiting hours because of delayed starting time of vaccination activities. Long waiting time is a challenge for every individual utilising public health services, regardless of citizenship (Malakoane et al. 2020). Many participants reported tolerating long waiting times at the healthcare facility because they have no choice but to wait for their turn. Willingness to wait for a specific service can vary depending on a variety of factors, such as the perceived value of the visit and the costs of a long wait at the healthcare facility (Chu et al. 2019). Child immunisation for immigrant society and refugee resettlement is required, more especially where they experience a lower immunisation coverage and a higher burden of vaccine-preventable childhood illness (Tankwanchi et al. 2021). White et al. (2020) report that migrants compare their expectations with practices in their home country, and may become dissatisfied if the time spent waiting is longer than what they have been used to. Chu et al. (2019), Egbujie et al. (2018) and Karat et al. (2022) highlight that informing patients beforehand of the possibility of waiting for healthcare services may reduce stress and frustration and promote tolerance.

Schmidt et al. (2018) write that the nature of migrants’ employment and financial constraints are classified as personal barriers influencing the accessibility of healthcare services and satisfaction. Migrant mothers in the current study reported that these two factors lead to delays in accessing healthcare facilities. It is evident from the research that migrant mothers need to be financially stable in order to pay for their travelling costs; otherwise they are delayed from seeking immunisation services because of the fear of losing their jobs, as they are also faced with challenges of finding employment. The study conducted by Lowane and Lebese (2021) shares similar findings with this study, reporting that migrants receive low wages and have to extend their working hours to generate sufficient income to finance their living costs, as they generally earn low wages.

Interpersonal relationships between migrant mothers and healthcare workers were identified as a factor that can either promote or inhibit the utilisation of immunisation services in healthcare facilities. The study participants reported both negative and positive experiences. Amukugo et al. (2020) indicated that poor interpersonal relationships negatively impact the delivery of healthcare and services. Most study participants mentioned that they were treated fairly and equally to local citizens. White et al. (2020) report that few participants mentioned being treated fairly during the healthcare visits.

Although most of the study participants reported that the healthcare workers were polite to them and treated them with a positive attitude, the comments of some participants reported a negative attitude towards them and feelings that they were discriminated against. Complaints about negative attitudes and discrimination because of nationality are of concern, and they have been reported by other studies as well (Mehdiyar et al. 2016; Munyewende & Nunu 2017). People subjected to perceived or actual discrimination often feel sadness, helplessness, anxiety, depression and low self-esteem. As a result, individuals become reluctant to access healthcare services and their quality of care becomes compromised (Gurrola & Ayón 2018). Despite facing diverse challenges and experiencing discrimination from healthcare workers, migrant mothers found a way to navigate and engage with the public healthcare sector to receive child vaccinations (Vearey et al. 2018). Crush and Tawodzera (2014) emphasise that discrimination is unacceptable, and healthcare managers must come up with a strategy to prevent healthcare system discrimination and to ensure that health providers observe and adhere to their professional and ethical obligations (Kuan et al. 2020; South African Nursing Council 2013).

### Limitations of this study

The study was conducted in one metropolitan municipality situated on the east coast of the Eastern Cape province, South Africa, at a point in time and cannot be generalised to the entire country. The sample size was comparatively small; hence, the experiences of migrant mothers in accessing immunisation services might not be representative of all migrant mothers with children in Buffalo City Metropolitan Municipality. Because most of the interviews were conducted in the healthcare facilities, participants in this study could have been hesitant to fully share their experiences for fear that healthcare staff might retaliate if they gave a negative review. In addition, the interviews were conducted in English, and this might have affected their ability to understand the questions well and to reply accurately.

The main strength of this study is the use of qualitative methods to generate in-depth views of migrant mothers. This study is one of the first surveys in South Africa to explore the utilisation of immunisation services provided in PHC facilities by migrant mothers. To overcome the potential non-response bias, probing during the interview was carried out, and participants were encouraged to comment freely.

## Conclusion

In conclusion, this study explored the experiences of migrant mothers in utilising immunisation services at various healthcare facilities. Some experiences were positive and facilitated the utilisation of immunisation services, whereas some were negative experiences that served as barriers to accessing immunisation services. Language difficulties were reported by migrant mothers. Effective communication does not only promote good interpersonal relationships, but results in well-balanced activities that regulate patients’ emotions, facilitate the identification of patients’ needs and promote quality care. Poor interpersonal relationships, such as discrimination and negative attitudes towards the migrant mothers seeking immunisation services in healthcare facilities, can affect the immunisation status of South Africa and its attempt to achieve the target of the Sustainable Development Goals (SDGs) by the year 2030. The risk of an individual child contracting a vaccine-preventable disease is higher if the child is not vaccinated in time, and this results in unnecessary hospitalisation and even disability or death.

## Recommendations

This study calls for strengthening interpersonal working conditions and ethics, as well as promoting the implementation of regulations and standards that will prevent discrimination against migrant communities at all levels of healthcare systems. Several recommendations for upcoming researchers, nongovernmental organisations, the South African government and migrant groups to improve access to PHC child vaccination services by migrant mothers can be proposed. Future researchers should focus on other provinces as well to assess the experience of migrants accessing PHC services. Research focusing on the experience of healthcare workers providing services to migrants may also be of value to improve the service provided.
